# Validation and Vindication: Enhancing the quality of Electronic Health Record (EHR) outcomes in a large prospective study biobank

**DOI:** 10.23889/ijpds.v1i1.365

**Published:** 2017-04-19

**Authors:** Sam Sansome, Iain Turnbull

**Affiliations:** 1 The University of Oxford

## Objectives

To date we have derived and linked over 1.5 million electronic health records (EHR) to over 280,000 of our biobank participants (across 10 diverse urban and rural geographical regions of China) through established morbidity and mortality registries, and by linkage to the China national Health Insurance (HI) system to identify disease outcomes.

To ensure accuracy, completeness and consistency of these outcomes we have developed data handling, processing, and validation procedures. 

## Approach

On a disease-by-disease basis we have developed multiple strategies varying from simple standardisation of disease description to ICD10, to a more comprehensive system of retrieval and examination of medical notes.

For those diseases chosen for more precise phenotyping, such as stroke, we send trained research technicians to hospitals where they retrieve clinical documents that correspond to the disease outcomes as reported by registries and HI agencies.

A Portable Validation Device (PVD) tablet has been developed to guide the medical notes gathering process and `validate' chosen disease events, by photographing key documents and ascertaining disease subtypes.

For diseases selected for further adjudication, medical specialists in China are given access to an internet based Case Adjudication System for clinical Events (i-CASE). The information captured by PVD is presented in i-CASE according to pre-specified clinical and procedural criteria.

At least 5% of all of data undergoes quality control assessments at both validation and adjudication stages in the UK.

## Results

Diseases causing the greatest burden in China (Cancer, Diabetes, COPD, Stroke and IHD) have all been standardised and subsequently validated using the PVD where appropriate. To date, we have retrieved over 45,000 hospital records through the PVD and, more recently, over 32,000 IHD and Stroke cases have been adjudicated through the i-CASE system.

Outcomes are categorised as `confirmed', `re-classified', `sub-typed' or `refuted' and are integrated back into the study dataset for future analysis, along with additional data gathered from the notes.

## Conclusion

The study provides a uniquely rich and powerful resource for investigating environmental and genetic determinants of chronic disease in the Chinese population. By treating disease types on a case-by-case basis we can both confirm their accuracy, and to link more precise clinical phenotypes to emerging data types, such as genetic and omics data. These innovations in disease validation and adjudication will enable the study to reach new insights into the aetiology of chronic disease to improve the health of the population in China and elsewhere.

